# Olanzapine induced reproductive toxicity in male rats

**DOI:** 10.1038/s41598-021-84235-4

**Published:** 2021-02-26

**Authors:** Cankız Mina Ardıç, Sinem Ilgın, Merve Baysal, A. Burak Karaduman, Volkan Kılıç, Gözde Aydoğan-Kılıç, Şeyda Uçarcan, Özlem Atlı-Eklioğlu

**Affiliations:** 1 Department of Pharmaceutical Toxicology, Faculty of Pharmacy, Anadolu University, 26470 Eskişehir, Turkey; 2 Department of Biology, Faculty of Science, Eskişehir Technical University, Eskisehir, Turkey

**Keywords:** Drug safety, Toxicology

## Abstract

Although it is reported that olanzapine (OLZ), which is an atypical antipsychotic drug, causes sexual dysfunction in men, it is noteworthy that there is not any study evaluating the toxic effects of OLZ on the male reproductive system. In the scope of this research, it was aimed to assess the reproductive toxic effects of OLZ by oral administration of 2.5, 5, or 10 mg/kg of it to male rats for 28 days. For this purpose, sperm concentration, motility and morphology, and DNA damage were determined, and histopathological examination of testis tissue was carried out in rats. Also, the levels of serum follicle-stimulating hormone (FSH), luteinizing hormone (LH), and testosterone, which play roles in the regulation of reproductive functions, and the levels of glutathione (GSH), catalase (CAT), superoxide dismutase (SOD) and malondialdehyde (MDA) which play roles in reproductive pathologies as oxidative stress biomarkers, were determined. According to the results, normal sperm morphology was decreased in 5 ve 10 mg/kg OLZ-administered groups, and pathological findings were evident in the testicular structure of the OLZ-administered group when compared with the control group. It was determined that serum LH, FSH, and testosterone levels were decreased in the OLZ-administered group. Also, decreases of GSH levels in testis tissue were determined and evaluated as the markers of the oxidative stress induced by OLZ in the testis. In conclusion, it was determined that reproductive toxic effects were induced in rats by OLZ administration. This pathology was accompanied by alterations of the hormone levels and testicular oxidative stress.

## Introduction

Antipsychotic drugs are used to treat schizophrenia, a chronic severe disease affecting 21 million people worldwide, and treat a manic episode of bipolar disorder^1,2^. Generally, typical antipsychotics have more adverse effects, while the second generation of these drugs, known as atypical ones, have a more favorable adverse effect profile. Sedation, orthostatic hypotension, anticholinergic effects, extrapyramidal symptoms, agranulocytosis, cardiac arrhythmias, hyperprolactinemia, and sexual dysfunction, are the known adverse effects of these drugs^3–5^.

Environmental toxicants, occupational exposures, and drug-related reproductive adverse effects are the crucial indicators of male infertility^6,7^. One hundred eighty-six million people have infertility, and 50% of the infertility cases are compromised of male-factor infertility cases worldwide^6,8^. It is known that the male reproductive system is a target of drug toxicity, and repeated exposures to antipsychotic drugs may disrupt sexual function, spermatogenesis process, and also epididymal maturation via modifying hormones of hypothalamic-pituitary and gonadal axis or non-hormonal mechanism in men^9,10^.

An atypical antipsychotic drug, olanzapine (OLZ), is one of the most prescribed for acute phase and maintenance of schizophrenia, acute treatment of manic, mixed episodes of bipolar disorder and, also maintenance of bipolar I disorder^2,11^. As a thiobenzodiazepine derivative, it shows affinity to dopaminergic (D1–D5), serotonergic (5HT2,3,6), muscarinic (subtypes 1–5), adrenergic (α1–2), and histaminergic (H1) binding regions^3,12–14^. OLZ was shown to be related to adverse effects such as amenorrhea, menstruation disorders, impotence, galactorrhea, and sexual dysfunctions accompanied by increased prolactin levels in female patients^15^. Increased prolactin levels may induce hypogonadism due to gonadotrophin-releasing hormone, luteinizing hormone, and testosterone inhibition and consequently cause delayed spermatogenesis, reduced semen quality and sperm motility, and morphological anomalies in testes tissue in both rats and schizophrenic patients^2,14,16,17^. Konarzewska et al.^18^ showed that patients treated with risperidone and olanzapine had higher prolactin levels. Both agents were found to be related to reproductive hormone disorders and sexual dysfunction. However, there has been no comprehensive study in which the possible reproductive toxicity of OLZ and potential underlying mechanisms were investigated.

Therefore, this study's main goal was to investigate the possible toxic effects of OLZ on the male reproductive system by determining sperm concentration, motility, morphology, and DNA damage and by performing the histopathological examination of testicular tissue. Furthermore, serum FSH, LH, and testosterone levels, which are the critical players in the regulation of reproductive functions, were determined, and glutathione (GSH), catalase (CAT), superoxide dismutase (SOD), and malondialdehyde (MDA) levels of testis tissue were evaluated as the biomarkers of oxidative stress which take an essential part in reproductive disorders.

## Materials and methods

### Materials

OLZ was a kind gift from IE Ulagay-Menarini Group (Istanbul, Turkey). Urethane for anesthesia was obtained from the Sigma-Aldrich Corporation, Missouri, USA. Testosterone, follicle-stimulating hormone (FSH), luteinizing hormone (LH), glutathione (GSH), and malondialdehyde (MDA) levels along with superoxide dismutase (SOD) and catalase (CAT) activities were determined by ELISA kits from Shanghai Sunred Biological Technology Co., Ltd. (Shanghai, China).

### Methods

Animals used (Wistar male rats, 10–12 weeks old, app. 300 g/each) were obtained from Anadolu University Research Center for Animal Experiments. The rats were housed in laboratory conditions under a 12/12 h light/dark cycle (light on at 7:00 a.m.) at an average temperature of 24 °C (± 2 °C) with ad libitum access to pure water and food. The experimental protocol was approved by the Local Ethical Committee on Animal Experimentation of Anadolu University, Eskisehir, Turkey (Approval Number: 2013-9). All experiments performed were following relevant guidelines and regulations. Also, the study was carried out in compliance with the ARRIVE guidelines. The Anadolu University Research Project Commission funded this study under grant number 1401S018.

Animal groups were randomly assigned as follows.

- Control group (C): animals received the vehicle, distilled water, orally for 28 days (n = 8).
- 2.5 mg kg^−1^ OLZ-administered group (OLZ-2.5): animals received 2.5 mg/kg dose OLZ orally for 28 days (n = 8).
- 5  mg kg^−1^ OLZ-administered group (OLZ-5): animals received 5 mg/kg dose OLZ orally for 28 days (n = 8).
- 10  mg kg^−1^ OLZ-administered group (OLZ-10): animals received 10 mg/kg dose OLZ orally for 28 days (n = 8).

The doses of OLZ were selected according to previous studies^13,19,20^ its antipsychotic activity was shown at the doses of 2.5, 5, and 10 mg/kg. Also, the frequently used clinical doses of OLZ are between 5–30 mg/day, and the initial doses of it are 5–10 mg/kg, and in a few days, the dose is increased to 10 mg/kg orally for schizophrenia^15^. All the doses we have chosen were following the guidelines extrapolating human doses to animal doses^21^. All drugs were administered in a volume of 1 mL/100 g by dissolving in distilled water. The treatment period was under the guideline OECD 407: Repeated dose oral toxicity study in rodents^22^.

At the end of 24 h after the last dose, the following procedures were applied to rats.

**Figure 1:**
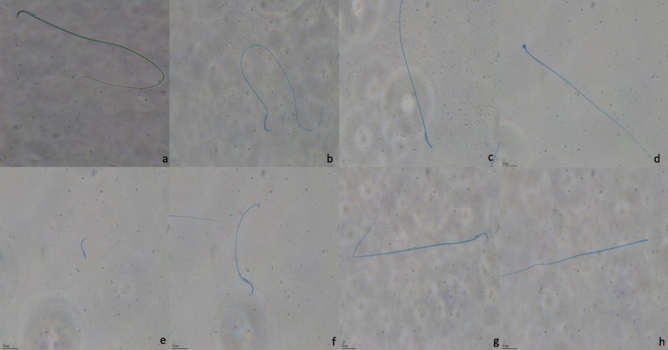
Sperm morphology observed under ×60 magnification following OLZ administration (**a**) Normal; head abnormalities; (**b**) bent neck; (**c**) banana; (**d**) amorphous; (**e**) Detached; (**f**) broken tail; (**g**) bent tail; (**h**) headless; tail abnormalities.

1. The rats were anesthetized by intraperitoneal injection of 1.5 g kg^−1^ urethane^23^.
2. The animals were euthanized via withdrawal of large volumes of blood from the heart. Blood samples were centrifuged for 15 min (at 4 °C and 1000×*g*) after overnight refrigeration (at 2–8 °C) and kept until hormonal analysis using ELISA kits.
3. The left testis and epididymis were cleaned of blood in phosphate-buffered solution (PBS) (8 g/L NaCl, 0.2 g/L KCl, 0.2 g/L KH_2_PO_4_, 1.14 g/L Na_2_HPO_4_, pH 7.4) and weighed to determine the relative organ weights.
4. The left epididymis was used to evaluate oxidative stress biomarkers. The left testis was divided and dry-freeze in liquid nitrogen and kept at – 20 °C. SOD, CAT activities, and GSH, MDA levels were measured using commercially available kits following the manufacturer's instructions.
5. Sperm DNA damage was evaluated by comet assay. Frosted microscope slides were covered with 1% normal melting point agarose in Ca^2+-^ and Mg^2+-^free phosphate-buffered saline. The sperm sample (10 μL) containing 1 × 10^5^ sperm/mL was suspended in 75 μL of 1% (w/v) low melting point agarose. 85 μL of this suspension was applied to the surface of the slide (pre-coated with 1% normal melting point agarose) to form a microgel and allowed to set at 4 °C for 5 min. The slide was dipped in cell lysis buffer (2.5 mol/L NaCl, 100 mmol/L EDTA, 10 mmol/L Tris–HCl, pH 10.0, containing 1% Triton X-100 added just before use and 40 mmol/L dithiothreitol) for 24 h at room temperature. Following the initial lysis, proteinase K was added to the lysis solution (0.5 mg/mL), and additional lysis was performed at 37 °C for 24 h. After cell lysis, the slide was washed three times with deionized water at 10 min-intervals to clear off salt and detergent from the microgels. The slide was placed in a horizontal electrophoresis equipment and allowed to equilibrate for 20 min with running buffer (500 mmol/L NaCl, 100 mmol/L Tris–HCl and 1 mmol/L EDTA, pH 9.0) before electrophoresis (0.60 V/cm, 250 mA) for 30 min. After electrophoresis, the slide was neutralized with 0.4 mol/l Tris (pH 7.5) and stained with SYBR Green I (1:10,000) (Sigma-Aldrich, Taufkirchen, Germany) for 1 h and covered with cover slips. This procedure was adopted by our laboratory from^30^ and performed as in our previous studies^23–26^.
6. The cauda of the right epididymis was used to evaluate the sperm parameters. Firstly, it was transferred to a Petri dish containing DMEM/Hams F-12 at 37 °C and cleaned blood vessels and fat. 0.5 cm of the cauda epididymis was removed and placed in another Petri dish containing 1 mL of the same medium, and sperms were allowed to swim out for 1 min^26–31^. Five μL of sperm cloud was placed on a Leja slide (Leja Products BV, Nieuw Vennep, Netherlands). Sperm concentration and motility were determined using by motility/concentration module of the Sperm Class Analyzer (SCA), version 5.4.0.1 (Microptic SL, Barcelona, Spain), at 50 frames/s.
7. Another five μL of the sperm cloud was placed on the clear end of a frosted slide by dragging the drop across the morphology analysis slide. The slide was dried before staining, and it was stained with Spermblue (Microptic Automatic Diagnostic System, Barcelona, Spain), according to Van der Horst and Maree (2009)^26–31^. The stained slide's morphology was evaluated by the morphometry module of Sperm Class Analyzer version 5.4.0.1 software. A total of 200 sperms/animal were analyzed randomly. Head and tail abnormalities of the sperms were detected according to previous criteria^32–36^. Sperms with a banana-shaped head, amorphous head, and bent neck and two-headed and headless sperms were counted for head abnormalities, whereas sperms with a bent tail and broken tail were counted for tail abnormalities (Fig. 1).
8. The right testis tissues were sliced into two mm^3^ and fixed in paraformaldehyde (4%) in phosphate buffer pH 7.2 for 2 h at 20–22 °C for histopathologic analysis. Tissue specimens were dehydrated in a graded series of alcohols and treated with a mixture of LR White (Electron Microscopy Sciences, Ft. Washington, PA) and ethanol (2:1) (v:v) for 1 h at room temperature. Tissue specimens were embedded in LR White and were sectioned at 700 nm (0.7 microns) using the Leica EM UC7 ultramicrotome. Semi-thin sections were stained with 1% toluidine blue/borax (pH 8.4) for 2 min and observed under a Leica DM 750 microscope equipped with a DFC camera. The same procedure was followed for  all testis sections (3 sections per animal)^37^. The five most circular seminiferous tubule cross-sections per animal were randomly selected for Stage IX of the seminiferous epithelium cycle. Epithelium height and tubule diameter were measured using the software Image J Software.
9. All data are expressed as the mean ± standard error. Statistical analyses of the groups were performed using the SigmaPlot Version 10 package program (Systat Software, San Jose, California, USA). To test normality, Shapiro–Wilk, and to test the equality of variance, the Levene’s test was used. In the sperm comet assay, a one-way analysis of variance (ANOVA) followed by Dunnett’s T3 test as a post-doc test was performed. In the other experiments, one-way ANOVA followed by Tukey’s test as a post-doc test was performed. P < 0.05 was considered statically significant.

## Results and discussion

### The evaluation of relative weights of testis and epididymis of rats

The weight alterations of organs and tissues are well-accepted as a sensitive marker of organ-related toxicities for a long time. In terms of reproductive toxicity, testis, epididymis, and seminal vesicle weights are determined in male reproductive studies^38,39^. To eliminate the differences between rats' body weights in the same groups, relative organ weight, which is defined as the ratio of organ weight to body weight/ is used^40^.

In a study of De Siqueira Bringel et al.^9^, the relative testis, epididymis, and prostate weights were shown to decrease in adult rats given 10 mg/kg OLZ i.p. for 45 days. However, according to our study results, relative testis and epididymis weight did not show any difference (Table 1). This finding can result from the secondary alterations (as also seen in the histopathological analysis of OLZ-treated rats) such as edema, which did not let relative organ weights differ from each other.

**Table 1:** Relative testis and epididymis weights among groups.

Groups	Relative testis weights (g/100 g body weight)	Relative epididymis weight (g/100 g body weight)
C	0.49 ± 0.05	0.20 ± 0.01
OLZ-2.5	0.48 ± 0.03	0.20 ± 0.01
OLZ-5	0.45 ± 0.03	0.19 ± 0.01
OLZ-10	0.46 ± 0.03	0.19 ± 0.01

Definition of abbreviations: *OLZ-2.5* 2.5 mg kg^−1^ olanzapine-administered rats for 28 days group, *OLZ-5* 5 mg kg^−1^ olanzapine-administered rats for 28 days group, *OLZ-10* 10 mg kg^−1^ olanzapine-administered rats for 28 days group. Results were given as mean ± standard error.

### The evaluation of sperm parameters

The criterion of semen quality are discussed according to male infertility, and generally, infertility is diagnosed by an abnormal semen analysis. Even though there is more than one parameter in sperm analysis, sperm count abnormalities such as oligospermia and azoospermia, sperm motility, and sperm morphology anomalies are evaluated as the most critical parameters in males^41,42^.

In a study of De Siqueira Bringel et al.^9^, the results of daily sperm production per testis and per gram did not show any difference from each other in adult rats who were administered 1, 2.5, 5, 10 mg/kg of OLZ for 45 days. Sperm morphology is well accepted as a sensitive marker of spermatogenesis quality and fertility. Also, sperms with morphological abnormalities can be accompanied by DNA damage, chromatin damages, and related pathologies clinically^43^.

The sperm quality parameters of our study are shown in Table 2. When OLZ-administered groups were compared with the control group in terms of sperm concentration, decreases were obtained. However, they were not statistically significant and also, in terms of motility, no significant difference was present. However, sperm morphology was shown to be worsened in OLZ-5 and OLZ-10 group when compared with the control and also with the low dose (OLZ-2.5) group.

**Table 2:** Sperm parameters of rats.

Groups	Sperm concentration (10^6^/mL)	Motility (%)	Normal sperm morphology (%)
C	1.67 ± 0.24	87.46 ± 3.15	84.00 ± 4.55
OLZ-2.5	1.32 ± 0.12	84.78 ± 6.43	77.16 ± 7.40
OLZ-5	1.40± 0.50	84.97 ± 5.17	64.86 ± 6.87^a,b^
OLZ-10	1.31 ± 0.32	84.45 ± 2.61	66.81 ± 5.00^a,b^

Definition of abbreviations: *OLZ-2.5* 2.5 mg kg^−1^ olanzapine-administered rats for 28 days group, *OLZ-5* 5 mg kg^−1^ olanzapine-administered rats for 28 days group, *OLZ-10* 10 mg kg^−1 ^ olanzapine-administered rats for 28 days group. Results were given as mean ± standard error.

All data were expressed as mean ± standard error.

^a^Different from control group (p < 0.05).

^b^Different from OLZ-2.5 (p ≤ 0.05).

The induction of the abnormal sperm morphology is thought to be caused by the damage of the genetic material of spermatogonia and spermatocyte or the injury during the spermatogenesis process in male patients^44,45^. According to our study results, abnormal sperm morphology, a sensitive biomarker of spermatogenesis quality and an indicator of fertility, was accepted as a marker of OLZ-induced reproductive toxicity.

Sperm DNA damage is also an independent measure of sperm quality and provides more precise diagnosis and prognostic information than standard semen analysis. Also, sperm DNA damage is a useful tool for determining male infertility because of the vulnerability of sperm to oxidation-related DNA damage and the lack of protection of the sperm nucleus against oxidative stress^46^.

Previous studies showed the induction of sperm DNA damage in males who were diagnosed as infertile. The source of DNA damage is generally multi-functional, and the sperm with DNA damage may be accepted as an independent marker of male subfertility from the parameters obtained from a standard semen analysis. Furthermore, the sperm samples of infertile men with normal standard semen parameters showed increased DNA damage. Sperm DNA fragmentation of more than 30% might cause adverse effects on reproductivity in male patients^45,47^.

**Figure 2:**
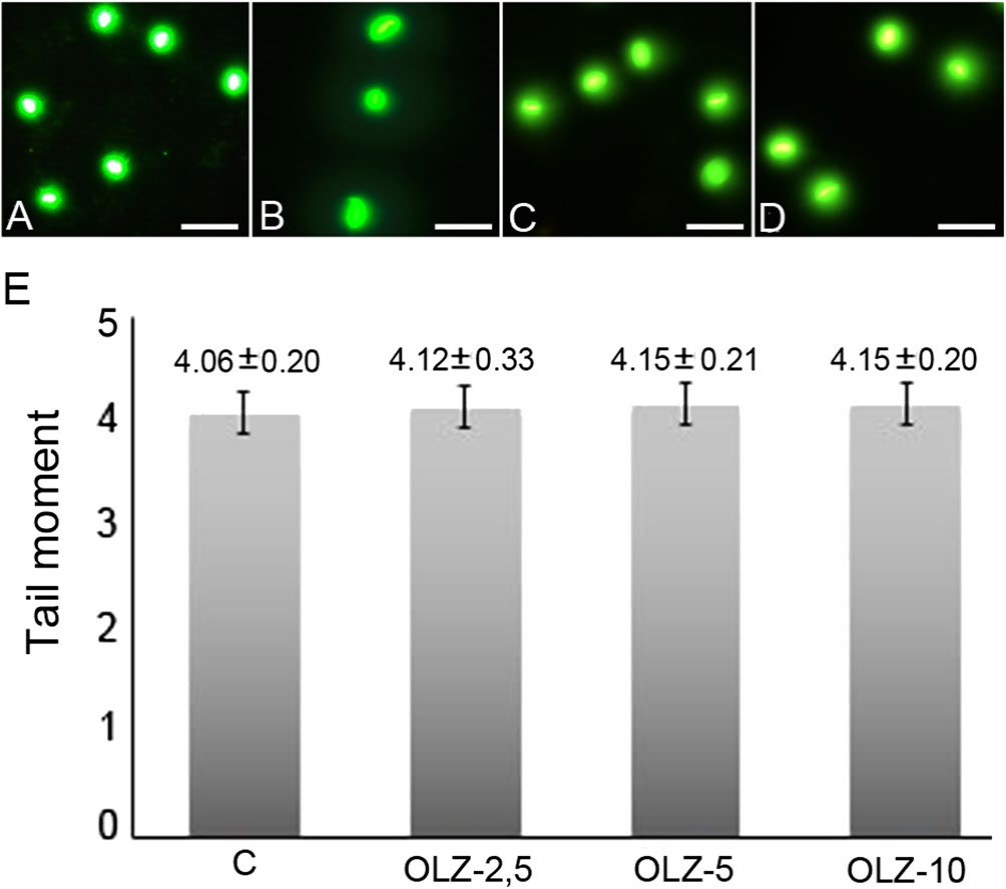
(**A–D**) Effect of olanzapine on the sperm DNA damage. (**A**) Sperm comet assay photo of control group (**B**) Sperm comet assay photo of OLZ-2.5 (**C**) Sperm comet assay photo of OLZ-5 (**D**) Sperm comet assay photo of OLZ-10. Stained with SYBR Green. Scale Bar:20 µm. (**E**) Sperm comet assay graphical illustration. No significant difference between groups in tail moment graph. Definition of abbreviations: *OLZ-2.5* 2.5 mg kg^−1^ olanzapine-administered rats for 28 days group, *OLZ-5* 5 mg kg^−1^ olanzapine-administered rats for 28 days group, *OLZ-10* 10 mg kg^−1^ olanzapine-administered rats for 28 days group. Results were given as mean ± standard error.

Comet test is a widely used sensitive method for detecting sperm DNA damage by determining broken DNA strands in various cell types^45,48^. It can detect the genotoxic potential of the drugs and their metabolites by interacting with their genetic material. For this reason, the comet test is suggested as a genotoxicity test for germ cells. Integration of tests comprising various sperm functions is needed because sperm function tests are not adequate to independently determine all of the germ cell toxicants. The parameters used to detect DNA damage in male rats are tail length, tail DNA percentage, and tail moment percentage. Among these, tail moment % is the most integrated parameter for evaluating the cell's general DNA damage^49^. The comet assay results are given in Fig. 2. According to these results, no significant differences were found among the groups in terms of the tail moment.

Sperm DNA damage might result from the abnormalities of chromatin packaging and separation^45^, oxidative stress, abnormal cell apoptosis, and hormonal deficiencies^49,50^. There have been conflicting results about the relationship between sperm DNA damage and sperm morphology in men^51^. In a previous study investigating the genotoxicity of OLZ, it was concluded that it did not have genotoxic potential in micronucleus and sister chromatid exchange tests on human lymphocytes^52^. In studies investigating the association of sperm parameters and DNA damage, there was no significant relationship between abnormal morphology and sperm DNA damage in men^51,53^. In another study, human sperms with typical morphologies; however, from infertile subjects were shown to have DNA fragmentation^54^. For this reason, significant increases in the abnormal sperm morphology in our study were not related to DNA damage.

### Histopathological examination of testis tissue

In regulatory toxicology studies, histopathological examination is accepted as one of the most sensitive biomarkers to detect toxicants' adverse reproductive effects^55^.

**Figure 3:**
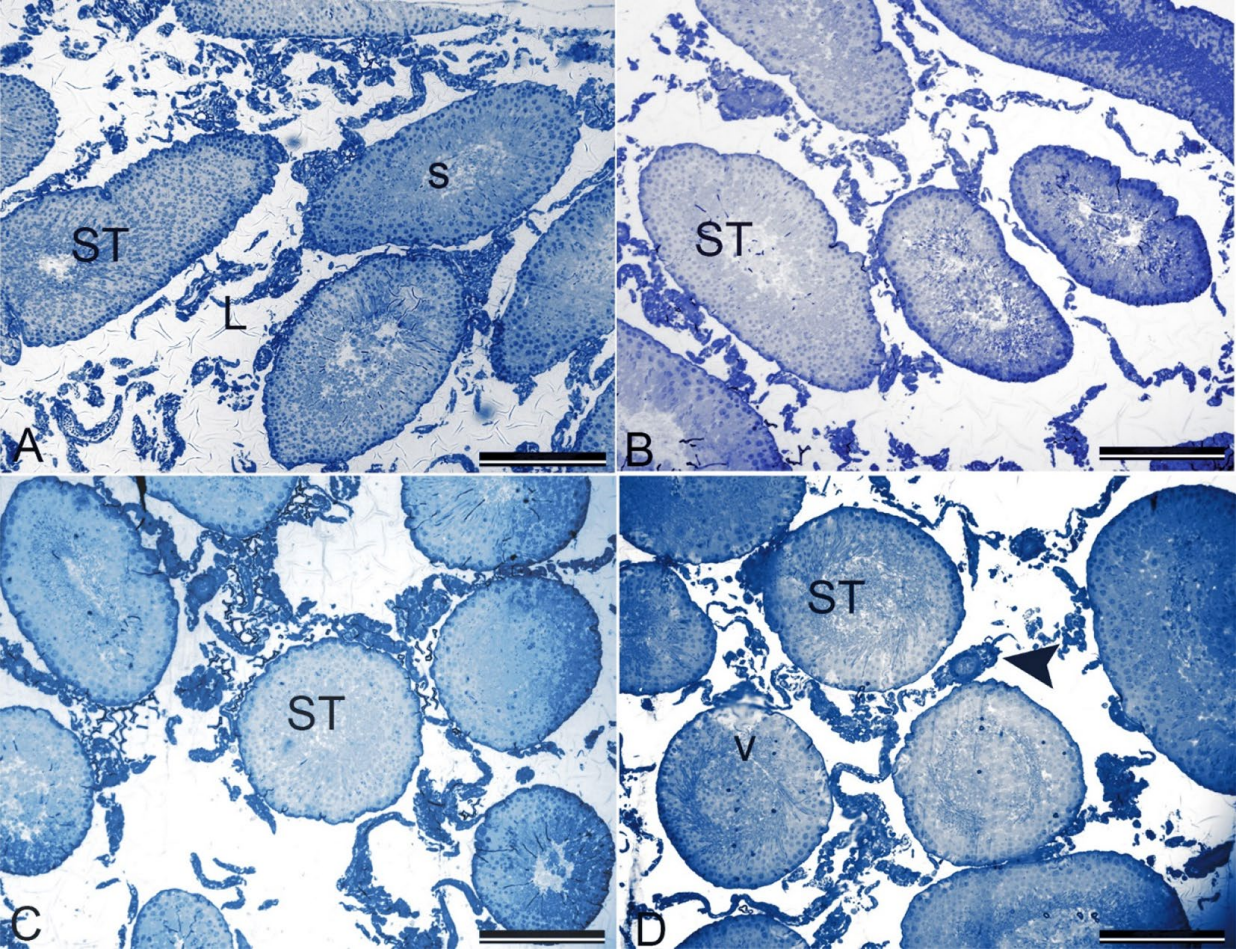
(**A–D**) Low magnification of seminiferous tubules and interstitial cells in control and olanzapine administrated animals (**A**) Control: Normal histology of-seminiferous tubules (ST), spermatozoa (s) and Leydig cells (L) (**B**,**C**) OLZ 2.5 and OLZ-5: No observable pathology in seminiferous tubules (ST). (**D**) OLZ-10: Large vacuoles (v) in seminiferous tubules (ST) and swelling of Leydig cells(arrowhead). 700 nm sections stained with toluidine blue. Scale Bar: 200 µm.

**Figure 4:**
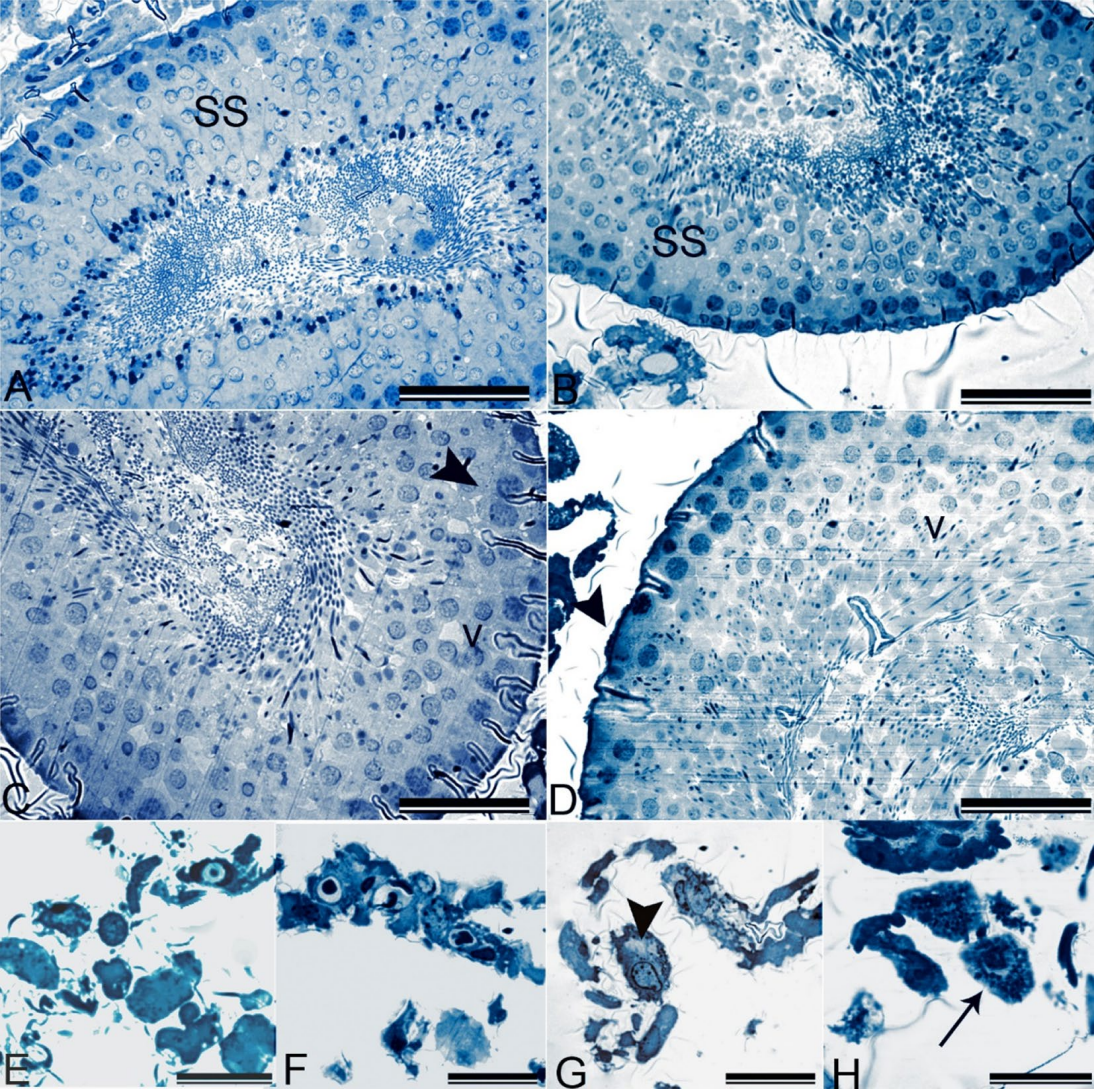
(**A–D**) High magnification of seminiferous tubules and interstitial cells in control and olanzapine administrated animals. (**A**) Control: Normal aspect of the seminiferous tubules containing regular cells of the spermatogenic series (SS) (**B**) OLZ-2.5: No observable pathology in spermatogenic series (SS). (**C**) OLZ-5: Swelling of spermatogenic cells (arrowhead) and mild intercellular vacuolation (v). (**D**) OLZ-10: Increased intercellular vacuolation (v), decreased sperm count and thickening of the basement membrane (arrowhead). 700 nm sections stained with toluidine blue. Scale Bar:50 µm. **(E**–**H)** High magnification of Leydig cells in control and olanzapine administrated animals. (**E**) Control: Normal appearance of Leydig cells (**F**) OLZ-2.5:No observable pathology in Leydig cells. (**G**) OLZ-5: Vacuolation (arrowhead) in Leydig cells. (**H**) OLZ-10: Swelling and increase in lipofuscin granules in Leydig cells (arrow). 700 nm sections stained with toluidine blue. Scale Bar:20 µm.

In our study, seminiferous tubule structures of the control group were found to be normal. Leydig cells in the interstitial area were also in the regular aspect and organization. Spermatogenic series and Sertoli cells in the tubules were also regular, and sperms were detected inside the lumen of seminiferous tubules (Figs. 3A, 4A, E).

There were no pathological appearances in the seminiferous tubules according to the histopathological sections of testis tissues of OLZ-2.5. Spermatogenic series, Sertoli cells, and Leydig cells were always in their usual appearance (Fig. 3B, 4B, F).

OLZ-5 group seminiferous tubules were also observed typically in low magnification. However, there was swelling in high magnification images of the spermatogenic cells. Mild vacuolation was the reversible pathological finding in the spermatogenic cells and Leydig cells in this group of animals (Fig. 3C, 4C, G).

In the OLZ-10 group, the vacuolization of seminiferous tubules became obvious. The swelling of Leydig cells and the increase of lipofuscin granules also attracted attention. An increase in lipofuscin granules represented lipid droplets' accumulation in lysosomes and was accepted as a differentiation marker in Leydig cells. There was an increase in vacuolization, reduction of sperm count, and thickening of the basement membrane (Fig. 3D, 4D,H). The most common indicator of Sertoli cell degeneration is vacuolization. Sometimes, vacuoles are separate and prominent; however, microvacuolization of Sertoli cell cytoplasm may also be seen in some cases. Generally, disorganization, exfoliation, or degeneration of germ cells accompany vacuolization and swelling. It is known that any functional deficit of Sertoli cells probably leads to germ cell degeneration by interacting with toxic substances^56,57^.

**Figure 5:**
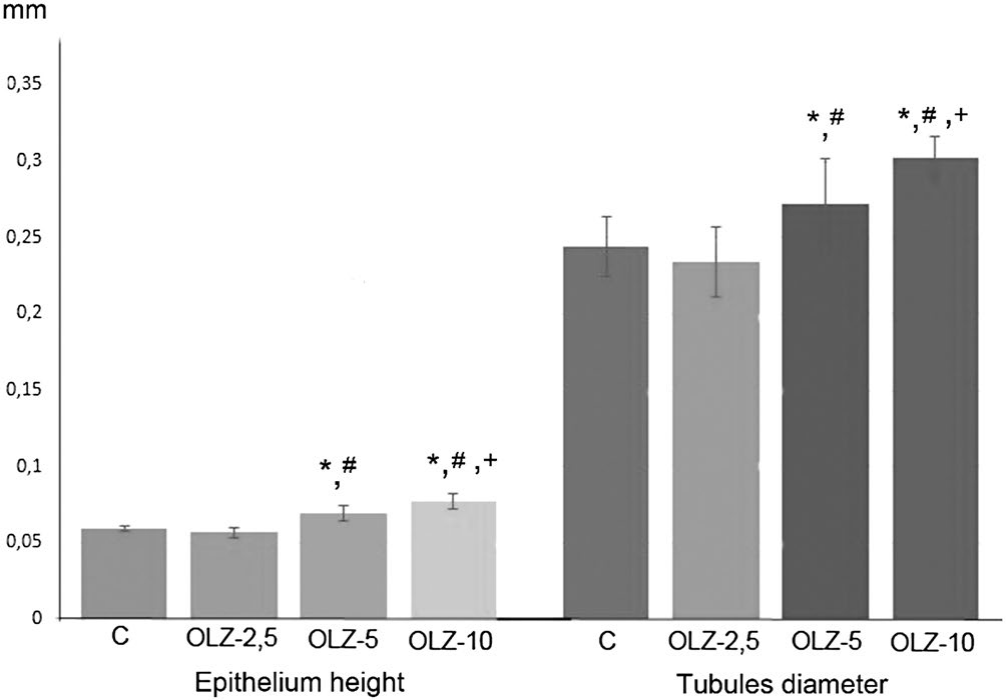
Morphometric analysis of the seminiferous epithelium for Stage IX of the seminiferous epithelium cycle. Data are presented as mean ± SEM, and the criteria for statistical significance were set at p < 0.05. *, Different from control; #, Different from OLZ-2.5; +, Different from OLZ-5.

In our study, OLZ-induced morphological abnormalities of sperms were also supported by histopathological findings and morphometric analysis presented in Fig. 5. Vacuolization, swelling, and the other related pathologies observed are the crucial indicators of OLZ-induced germ cell damage or reproductive toxicity, in other words, according to the histopathological analysis.

In a previous study, the histopathological analysis of testis tissues of rats treated with 5 and 10 mg/kg of OLZ revealed that desquamated germ cells in seminiferous tubules, giant cells with multiple nuclei, and intracytoplasmic vacuolization of Sertoli cells in testicular parenchyma. This finding was related to OLZ-induced testicular degeneration and the decrease of plasma testosterone levels. On the other hand, the decrease of plasma testosterone levels was associated with OLZ administration and a possible increase in prolactin levels^14^.

The histopathological findings of this study showed the OLZ-induced dose-dependent testis toxicity. These abnormalities also caused a decrease in sperm quality, according to our sperm morphology analysis.

### The evaluation of serum hormone levels

The hormone levels of the groups were shown in Table 3. According to the results, the FSH levels of OLZ-administered groups were significantly lower than the control group. Additionally, the FSH levels of the OLZ-10 group were also significantly reduced compared to the OLZ-2.5 group.

**Table 3:** Serum hormone levels of rats.

Groups	FSH (IU/L)	LH (mIU/mL)	Testosterone (pg/mL)
C	25.55 ± 1.24	15.86 ± 0.84	619.58 ± 95.10
OLZ-2.5	22.97 ± 1.32^a^	13.07 ± 1.60^a^	482.63 ± 107.86^a^
OLZ-5	20.57 ± 1.70^a^	13.32 ± 1.63^a^	449.18 ± 63.87^a^
OLZ-10	20.07 ± 1.73^a,b^	13.63 ± 1.52^a^	435.99 ± 88.63^a^

Definition of abbreviations: *OLZ-2.5* 2.5 mg kg^−1^ olanzapine-administered rats for 28 days group, *OLZ-5* 5 mg kg^−1^ olanzapine-administered rats for 28 days group, *OLZ-10* 10 mg kg^−1^ olanzapine-administered rats for 28 days group. Results were given as mean ± standard error.

^a^Different from control group (p < 0.05).

^b^Different from OLZ-2.5 (p < 0.05).

Serum LH levels were also decreased significantly in OLZ-administered groups when compared with the control group. Likewise, testosterone levels were also decreased significantly in the OLZ-administered group when compared with the control.

The significant decreases in testosterone levels might be the direct result of Leydig cells' degeneration, which are the center of testis androgen biosynthesis in adult rats^58^. Leydig cell atrophy was associated with the decrease of testosterone synthesis, and for this reason, the inhibition of testosterone is also frequently associated with the characteristic morphological differences of seminiferous tubules in both animals and humans^56,57^. The tubules contain higher testosterone and androgen binding protein levels in the seventh and eighth spermatogenesis cycle. In these cycles, Sertoli cell function and development of germ cells are dependent on sufficient testosterone levels. If testosterone synthesis decreases, these cells will undergo apoptosis in higher percentages and lead to spermatogenesis deficiencies^57^. Also, it was shown that OLZ was selectively toxic to Leydig cells in the histopathological examination. The alterations of LH levels were associated with degeneration of seminiferous tubules and germ cell deficiency, leading to a decrease of sperm production, spermatid count, and increase of sperm abnormalities in adult rats^58^. Atrophy and degeneration of seminiferous tubules, which contain Leydig and Sertoli cells, might be accompanied by the decrease of FSH levels^58^. It was shown that the decrease of gonadotrophins (FSH and LH) might lead to secondary hypogonadism and the decrease of testosterone levels have a negative impact on reproductive performance, social behaviors, and secondary sex characteristics in men^10^. According to our study results, the decreases of testosterone, FSH, and LH levels were supported and correlated with Leydig and Sertoli cells' vacuolization and deformations, which were found in the histopathological analysis and also with the abnormal sperm morphology. At this point, it is a fact that OLZ-induced decreases of testosterone, FSH, and LH might harm germ cell development, sperm quality, reproductive performance, and fertility.

When the limited number of studies investigating OLZ and related hormonal changes were reviewed, OLZ was administered 4 mg/kg with 2 mg/kg risperidone, and FSH levels were found to be decreased in adult male rats^59^. In another study, 10 mg/kg OLZ was administered to adult male rats for 45 days; testosterone levels were decreased significantly^14^. In previous epidemiological studies in which OLZ 10–20 mg/kg/day-treated patients for 4–12 days were reported to have hyperprolactinemia and sexual disorders^18^.

OLZ-induced hyperprolactinemia leads to hypogonadism-related galactorrhea, sexual dysfunction, gynecomastia, impotence, and decreased bone density were reported in men^15^. Most antipsychotics block D2 receptors; hence dopamine in the central nervous system causing hyperprolactinemia, suppressing hypothalamic–pituitary–gonadal axis and GnRH release secondarily decrease of testosterone, FSH, and LH levels might be determined^15,60^. Previous studies showed that OLZ caused an increase of prolactin levels and, consequently, might lead to a delay in spermatogenesis, reduced sperm motility and quality, and caused morphological abnormalities in the testis in both men and adult rats^14,61^. Therefore, OLZ-induced reductions of testosterone, FSH, and LH levels might result from OLZ-induced hyperprolactinemia.

### The evaluation of oxidative stress in samples

It was found that reactive oxygen species (ROS) are increased by 25% of infertile males. Lower ROS levels are sufficient for normal sperm function; however, increases in sperm ROS levels caused spermatozoa defects and sperm dysfunction^50^. In mature spermatozoa, ROS plays an essential role in capacitance, acrosome reaction, mitochondrial sheath stability, and sperm motility^62^. It is known that spermatozoa are susceptible to ROS because the sperm cell membrane contains higher levels of unsaturated fatty acids, and the cytoplasm contains low levels of enzymes that neutralize ROS^63^. The loss of cell membrane integrity, increase of cell membrane permeability, inactivation of cellular enzymes, DNA damage, and cell apoptosis might be caused by lipid oxidation^62^. As a result, decreased sperm count, activity and motility, and abnormal sperm morphology might occur^63^.

The SOD, CAT, GSH, MDA levels of our groups were presented in Table 4. According to these results, CAT and MDA levels of OLZ-administered groups were indifferent from the control group. In terms of testis GSH levels, the GSH levels of the OLZ-10 group were significantly lower than the control group. According to the SOD levels of groups, SOD activity was increased significantly in OLZ-5 and OLZ-10 groups when compared with the control group. Additionally, the SOD activity of the OLZ-10 group was significantly higher than the OLZ-2.5 group.

**Table 4:** The levels of oxidative stress biomarkers in rat’s testes.

Groups	CAT (ng/mL)	GSH (ng/mL)	MDA (mcg/mL)	SOD (ng/mL)
C	123.64 ± 9.64	432.45 ± 24.99	8.72 ± 0.52	29.96 ± 1.59
OLA-2.5	107.67 ± 17.02	388.01 ± 89.31	8.51 ± 3.27	33.43 ± 2.59
OLA-5	116.82 ± 17.27	373.50 ± 98.70	8.02 ± 1.05	35.15 ± 3.45^a^
OLA-10	112.69 ± 25.47	334.31 ± 55.35^a^	9.03 ± 1.07	37.04 ± 2.27^a,b^

Definition of abbreviations: *OLZ-2.5* 2.5 mg kg^−1^ olanzapine-administered rats for 28 days group, *OLZ-5* 5 mg kg^−1^ olanzapine-administered rats for 28 days group, *OLZ-10* 10 mg kg^−1^olanzapine-administered rats for 28 days group. Results were given as mean ± standard error.

All data were expressed as mean ± standard error.

^a^Different from control group (p < 0.05).

^b^Different from OLZ-2.5 (p ≤ 0.05).

The free radicals' role inducing sperm oxidative stress has a higher impact on weak sperm function and induction of infertility. ROS's negative impact on sperm function and motility was linked to higher ROS and RNS levels in rat testis^64^. An increase of ROS levels or the alterations of prooxidants to oxidants can cause oxidative stress in semen. Also, the decreases in antioxidant activity in semen were associated with idiopathic infertility. It is possible that the increase of ROS production inhibits antioxidant enzymes, or naturally decreased levels of these antioxidant enzyme expressions might cause oxidative stress. Consequently, lipid peroxidation increases, sperm motility, viability, and functionality decreases, leading to infertility^65^.

In our study, the decrease of GSH levels in OLZ administered groups was an indicator of oxidative stress. ROS may alter antioxidant defense mechanisms by decreasing GSH concentrations^56^. In previous studies, oxidative stress was related to the significant increase of lipid peroxidation and decreased GSH levels in adult male rats^66,67^. In a study of Tremellen, a decrease of GSH concentration sufficient to maintain GPx activity was shown to cause adverse conditions such as semen redox homeostasis exposed to oxidative stress in men^68^. At this point, this finding also supports histopathological alterations in the highest OLZ dose group. Increased oxidative stress and acutely GSH levels decrease, whereas antioxidant enzyme levels increase to struggle with ROS^69^. In our study, a dose-dependent increase of SOD was considered an acute result of increased oxidative stress and a rapid stimulation of SOD to battle for ROS. Additionally, there are more supportive findings showing that SOD increase occurs as a result of ROS increase. Superoxide anion is increased in infertility cases or low sperm quality in men^70,71^. In a study by Togar et al.^46^, higher doses of OLZ were shown to increase human cells' oxidative stress. In conclusion, abnormal sperm morphology in the high dose OLZ administered group and the testicular structure's degenerative findings were associated with the OLZ-induced oxidative stress.

## Conclusion

Schizophrenia is seen worldwide, having symptoms like hallucinations, introversion, and delirium; however, there is still insufficient knowledge of its etiology. The initiation of this disease 80% occurs at the beginning of the reproductive period, and it affects neuroendocrine parts of the brain and causes alterations of reproductive functions. The antipsychotic drugs used in the treatment of schizophrenia directly affect hormonal regulation or indirectly induce sexual dysfunction, disruption of the spermatogenesis process, and dysregulation of epididymal maturation, thereby causing reproductive toxic effects. In this context, it was aimed to investigate the reproductive toxic effects of OLZ, one of the most prescribed, on male rats in this study, which was performed independently of other risk factors that can affect the reproductive system. According to this study results, OLZ reduced normal sperm morphology, induced toxicity on testes tissue dose-dependently. The underlying mechanisms were the increased oxidative stress, the particular damage on Leydig cells, and the disruption of the hypothalamic-pituitary and gonadal axis characterized by FSH and LH decreases. Our study is the first study that investigated OLZ-induced reproductive toxicity via various biomarkers. Based on the results of this study, further epidemiological studies are needed. Sperm parameters and reproductive hormone levels may be suggested to be monitored in the patients under OLZ treatment before and after the treatment process to determine the toxicity risk related to OLZ.
